# Baicalin, Amoxicillin, and Probenecid Provide Protection in Mice Against *Glaesserella parasuis* Challenge

**DOI:** 10.3390/biom15040507

**Published:** 2025-03-31

**Authors:** Jingyang Li, Ronghui Luo, Yunjian Fu, Siyu Liu, Qiaoli Dong, Yamin Sun, Xinyue Tian, Yi Zhu, Peiyi Wang, Ling Guo, Qirong Lu, Chun Ye, Shulin Fu, Yinsheng Qiu

**Affiliations:** 1 Hubei Key Laboratory of Animal Nutrition and Feed Science, Wuhan Polytechnic University, Wuhan 430023, China; lijingyang026@163.com (J.L.); 18971562947@163.com (R.L.); mopanfyj@163.com (Y.F.); lusy617@163.com (S.L.); 15838785140@163.com (Q.D.); 18137892679@163.com (Y.S.); tianxinyue111@126.com (X.T.); 18272347532@163.com (Y.Z.); peiyi1220@163.com (P.W.); guoling1101@whpu.edu.cn (L.G.); qirongluvet@whpu.edu.cn (Q.L.); yechun@whpu.edu.cn (C.Y.); 2 Hubei Collaborative Innovation Center for Animal Nutrition and Feed Safety, Wuhan 430023, China

**Keywords:** baicalin, amoxicillin, probenecid, *Glaesserella parasuis*, anti-inflammatory, antibacterial

## Abstract

*Glaesserella parasuis* (*G. parasuis*) causes Glässer’s disease and systemic inflammatory responses in the host. The currently available therapies have limited efficacy and fail to achieve a balance between anti-inflammatory and antibacterial effects. In this study, we investigated the effects of baicalin, amoxicillin, and probenecid on blood biochemical parameters, routine blood indicators, survival rate, bacterial burden, and pathological tissue damage in *G. parasuis*-challenged mice. Treatment with baicalin, amoxicillin, and probenecid significantly modified the blood biochemical parameters and routine blood test indicators, increased the survival rate, attenuated the bacterial burden, and alleviated pathological tissue damage in *G. parasuis*-challenged mice. Treatment with baicalin, amoxicillin, and probenecid also increased the number of CD3^+^, CD3^+^CD4^+^, and CD3^+^CD8^+^ T cells as measured by flow cytometry, and restored the intensity of the CD3, CD4, and CD8 protein expression in the blood vessels of *G. parasuis*-challenged mice by immunohistochemistry. These compounds reduced interleukin 1β (IL-1β), IL-18, tumor necrosis factor alpha (TNF-α), and high mobility group box 1 protein (HMGB1) expression in the spleen of *G. parasuis*-challenged mice. Furthermore, baicalin, amoxicillin, and probenecid inhibited activation of the family pyrin domain containing 3 (NLRP3) inflammasome and apoptosis in the spleen of *G. parasuis*-challenged mice. This study showed the important roles of baicalin, amoxicillin, and probenecid in the modulation of the inflammatory response of Glässer’s disease. The findings might provide new strategies for combination therapy using antibiotics and anti-inflammatory drugs to control *G. parasuis* infection.

## 1. Introduction

*Glaesserella parasuis* (*G. parasuis*), one of the most common bacterial upper respiratory pathogens, is a serious threat to porcine industry development [1]. *G. parasuis* is responsible for Glässer’s disease of the pig and could lead to huge economic losses [2]. The typical clinical signs of Glässer’s disease are fibrinous polyserositis, polyarthritis, and meningitis [3]. To date, 15 serotypes of *G. parasuis* have been verified by the gel immunodiffusion test, but up to 20% of the isolates cannot be classified [4]. Serovar 5 is thought to be the highly virulent and epidemic strain in China [5]. So far, the pathogenic mechanism of *G. parasuis* remains unclear. Previous research has shown that the *htrA* gene is involved in adhesion and related to survival and pathogenicity of *G. parasuis* [6]. PilA of *G. parasuis* mediates adhesion to porcine kidney-15 (PK-15) cells and swine tracheal epithelial cells (STECs) through fibronectin and laminin [7]. Deletion of QseBC weakens the virulence of *G. parasuis*, adhesion to pulmonary alveolar macrophage (PAM) cells, and host inflammation [8]. Due to the unclear pathogenesis of *G. parasuis*, it is important to study its infection mechanisms to identify effective treatments.

In clinical practice, antibiotics are generally used to control bacterial infections in pigs [9]. Amoxicillin belongs to the aminopenicillin family of penicillin; it can inhibit the synthesis of bacterial cell walls and has efficient broad-spectrum antibacterial effects [10]. *G. parasuis* is sensitive to amoxicillin; thus, it has been widely used to control *G. parasuis* infection on pig farms [11]. However, the clinical management of *G. parasuis* infections is increasingly constrained by emerging antimicrobial resistance [12], which underscores the critical need for implementing combination therapeutic strategies to optimize antimicrobial stewardship while maintaining clinical efficacy. In the present study, amoxicillin was used as the positive control.

Probenecid is lipid soluble and used as a putative neurotherapeutic [13]. Probenecid has been shown to have an anti-inflammatory function via inhibiting OAT1/3 mediated transport of kynurenic acid in the brain [14]. It can modulate myocardial contractility and displays anti-inflammatory properties that might inhibit inflammasomes, modify purinergic-pannexin-1 signaling and TRPV2 channels [15]. In the previous study, mice were used to establish the *G. parasuis* infection model [16]. However, the anti-inflammatory efficacy of probenecid in *G. parasuis*-challenged mice has not been investigated.

Due to the excessive use of antibiotics, the phenomenon of antibiotic resistance has become increasingly severe [17]. Traditional Chinese medicine could alleviate the problem of antibiotic resistance. Baicalin, extracted from *Scutellaria baicalensis* Georgi, has important anti-inflammatory [18] and anti-microbial effects [19]. Baicalin suppresses interleukin-1β (IL-1β)-induced apoptosis, inflammation, oxidative stress, and extracellular matrix degradation in human nucleus pulposus cells [20]. Baicalin reduces renal inflammation in mesangial proliferative glomerulonephritis through the nuclear factor erythroid 2-related factor 2 (Nrf2)/antioxidant response element (ARE) and phosphoinositide 3-kinase (PI3K)/AKT pathways [21]. Baicalin relieves lung inflammation triggered by lipopolysaccharide (LPS) in mice via the nuclear factor kappa B (NF-κB) and mitogen-activated protein kinase (MAPK) pathways [22]. However, there has been no investigation of antibiotic and traditional Chinese medicine combination therapy to control *G. parasuis* infection. Therefore, in this study, we investigated the ability of amoxicillin, probenecid, the combination of amoxicillin and probenecid, and baicalin to ameliorate *G. parasuis* infection in mice.

## 2. Results

### 2.1. The Effects of Baicalin, Probenecid, Amoxicillin, and the Combination of Probenecid and Amoxicillin on the Body Weight (BW), Survival Rate, and Bacterial Burden of G. parasuis-Challenged Mice

**Figure 1:**
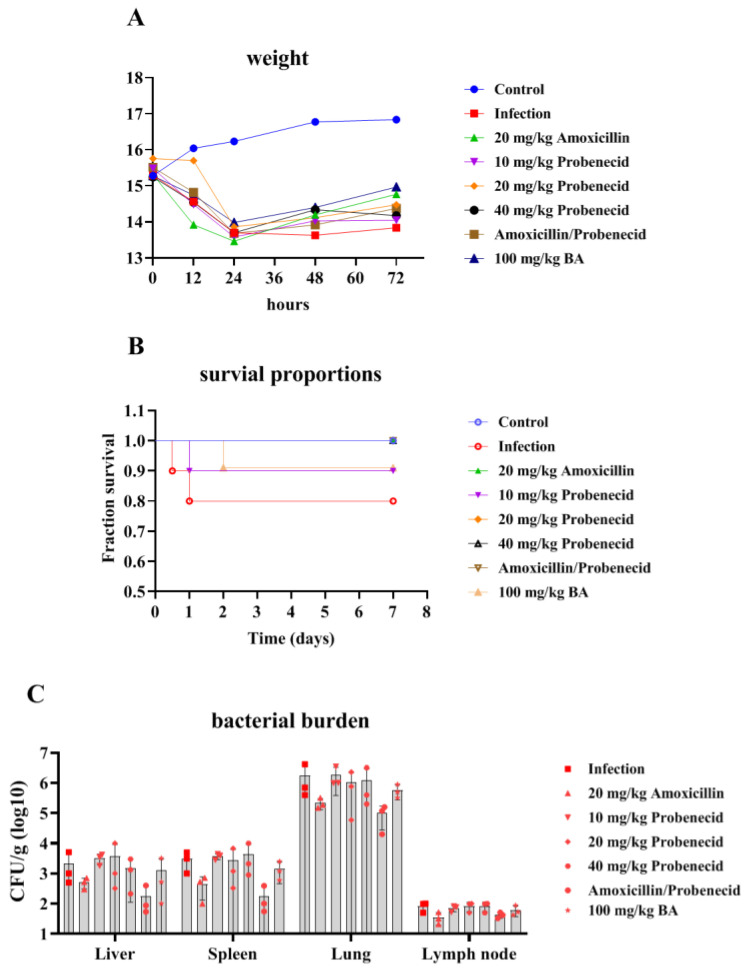
Detection of the effects of baicalin on the body weight (**A**), survival rate (**B**), and bacterial burden (**C**) of *G. parasuis*-challenged mice. When the mice were pretreated with drugs and challenged by *G. parasuis,* the body weight, survival rate, and the bacterial burdens in the liver, spleen, lung, and lymph node were determined.

After *G. parasuis* challenge, the weight of the mice from the infection, Pro10, Pro20, Pro40, Amo20, the Pro20 + Amo20, and the Bai100 groups decreased (Figure 1A; see Section 4.4 for details on the groups). The mice from the control group showed an increase in BW, and the mice from the infection group grew the slowest (Figure 1A). Mice treated with baicalin exhibited the fastest BW recovery (Figure 1A).

Following challenged with 2 × 10^9^ colony-forming units (CFUs) of *G. parasuis,* the infection group showed a mortality rate of 20% (Figure 1B). The Bai100 group provided 90% protection against the *G. parasuis* challenge compared to the infection group (*p* < 0.05) (Figure 1B). The Pro10, Pro20, Pro40, Amo20, and Pro20 + Amo20 groups provided 90%, 100%, 100%, 100%, and 100% protection, respectively, when the mice were challenged by *G. parasuis* (Figure 1B). The Pro10, Pro20, Pro40, Amo20, and Pro20 + Amo20 groups showed a higher survival rate than the infection group (*p* < 0.05) (Figure 1B). Furthermore, the Pro20, Pro40, Amo20, and Pro20 + Amo20 groups showed a higher survival rate than the Pro10 and Bai100 groups (*p* < 0.05) (Figure 1B).

We examined the bacterial burden in the liver, spleen, lung, and lymph node. The moribund mice from the infection group had detectable bacteria in the liver, spleen, lung, and lymph node. The surviving mice had a variable bacterial burden in the tissue specimens. The surviving mice from the Amo20 and Pro20 + Amo20 groups had less bacterial burden in the liver, spleen, lung, and lymph node (Figure 1C). The mice from the Pro10 group had a higher bacterial burden in the lung (Figure 1C).

### 2.2. Baicalin, Probenecid, Amoxicillin, and the Combination of Probenecid and Amoxicillin Altered the Blood Biochemical Parameters and Routine Blood Test Indicators in G. parasuis-Challenged Mice

We explored the blood biochemical parameters and routine blood test indicators 12, 24, and 72 h after challenging mice with *G. parasuis*. Total bilirubin (TB), alanine aminotransferase (ALT), cholesterol (CHOL), and triglycerides (TG) were significantly upregulated, and albumin (ALB), alkaline phosphatase (ALP), glucose (GLU), calcium (Ca), and lactate dehydrogenase (LDH) were decreased at 12, 24, and 72 h after *G. parasuis* challenge in the infection group compared with the control group (*p* < 0.05) (Table 1, Table 2 and Table 3). We found that 20 mg/kg BW amoxicillin reduced the ALT and CHOL levels and increased the Ca level 12 h after the *G. parasuis* challenge compared with the infection group (*p* < 0.01) (Table 1). Treatment with 20 or 40 mg/kg BW probenecid or 20 mg/kg BW probenecid and amoxicillin increased the ALB and LDH level and reduced the phosphorus (P) level compared with the infection group (*p* < 0.05) (Table 1). When the mice were pretreated with 100 mg/kg BW baicalin for 12 h, the CHOL and P levels were decreased compared with the infection group (*p* < 0.05) (Table 1). At 72 h after *G. parasuis* challenge, the aspartate aminotransferase (AST), CHOL, TG, and high-density lipoprotein (HDL) levels had returned to normal levels (Table 3).

Regarding the routine blood test results, the white blood cell (WBC), red blood cell (RBC), platelet (PLT), and lymphocyte (LYM) levels were significantly lower in the infection group compared with the control group at 12, 24, and 72 h after *G. parasuis* challenge (*p* < 0.05) (Supplemental Tables S1–S3). Compared with the control group, neutrophils (NE) were significantly increased in the infection group at 24 h after the *G. parasuis* challenge (*p* < 0.001) (Supplemental Table S2) and then decreased at 72 h (*p* < 0.001) (Supplemental Table S3). Treatment with 20 mg/kg BW amoxicillin increased the PLT and monocyte (MONO) levels at 12 h after the *G. parasuis* challenge compared with the infection group (*p* < 0.01) (Supplemental Table S2). The RBC, MONO, and eosinophil (EOS) levels at 12 h after the *G. parasuis* challenge were significantly higher in the Pro40, Pro20+Pro20, and Bai100 groups compared with the infection group (*p* < 0.05) (Supplemental Table S2). At 72 h after the *G. parasuis* challenge, most of the routine blood indicators had returned to normal levels (Supplemental Table S3).

### 2.3. Baicalin, Probenecid, Amoxicillin, and the Combination of Probenecid and Amoxicillin Inhibited Inflammatory Cytokines and High Mobility Group Box 1 Protein (HMGB1) Production in G. parasuis-Challenged Mice

**Figure 2:**
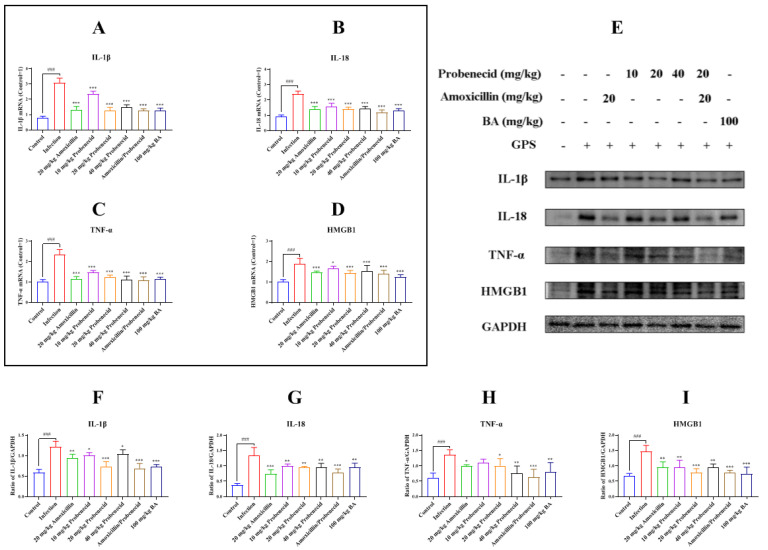
Measurement of the inflammatory cytokines and HMGB1 production in *G. parasuis*-challenged mice. After the mice were challenged, the spleens were collected and IL-1β (**A**,**E**,**F**), IL-18 (**B**,**E**,**G**), TNF-α (**C**,**E**,**H**), and HMGB1 (**D**,**E**,**I**) expressions of mRNA and protein levels were determined by RT-PCR and Western blotting. ^###^ *p* < 0.001 versus control group; * *p* < 0.05, ** *p* < 0.01, and *** *p* < 0.001 versus the infection group.

We evaluated the expression of the inflammatory cytokines IL-1β, IL-18, and tumor necrosis factor α (TNF-α) after mice were challenged by *G. parasuis* for 72 h by using quantitative real-time reverse transcription PCR (RT-qPCR) and Western blotting. *G. parasuis* infection increased IL-1β, IL-18, and TNF-α messenger RNA (mRNA) levels compared with the control group (*p* < 0.001) (Figure 2A–C). The IL-1β, IL-18, and TNF-α mRNA levels were decreased in the Pro10, Pro20, Pro40, Amo20, Pro20 + Amo20, and Bai100 groups compared with the infection group (*p* < 0.001) (Figure 2A–C). The IL-1β, IL-18, and TNF-α protein levels were increased in the infection group compared with the control group (*p* < 0.001) (Figure 2E–H). The IL-1β, IL-18, and TNF-α protein levels were reduced in the Pro10, Pro20, Pro40, Amo20, Pro20 + Amo20, and Bai100 groups compared with the infection group (*p* < 0.05) (Figure 2E–H) (except TNF-α in the Pro10 group).

The HMGB1 mRNA and protein levels in the spleen were increased in the infection group compared with the control group (*p* < 0.001) (Figure 2D). The HMGB1 mRNA and protein levels were decreased in the Pro10, Pro20, Pro40, Amo20, Pro20 + Amo20, and Bai100 groups compared with the infection group (*p* < 0.05) (Figure 2D,E).

### 2.4. Baicalin, Probenecid, Amoxicillin, and the Combination of Probenecid and Amoxicillin Modified the Proportion of CD3^+^, CD4^+^, and CD8^+^ T Cells in the Blood and Spleen of G. parasuis-Challenged Mice

**Figure 3:**
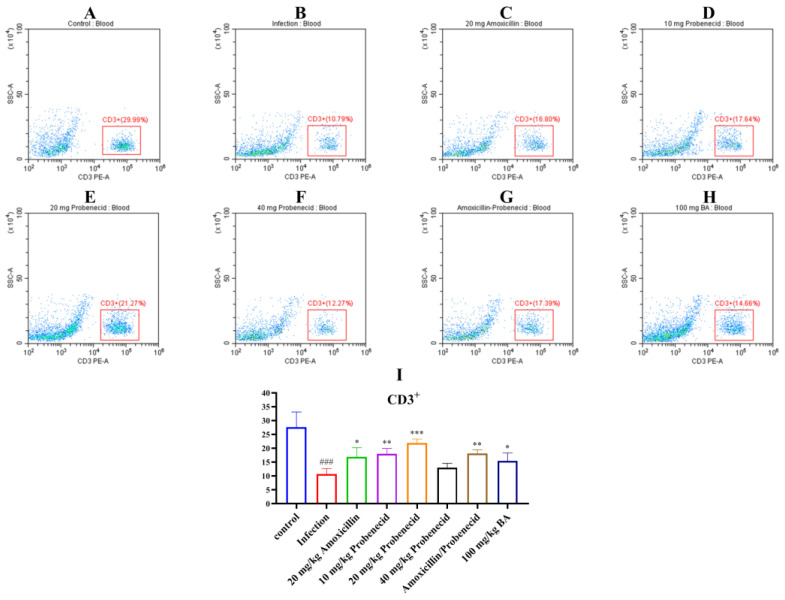
Flow immune cells of CD3^+^ T cells from the blood were analyzed using cytometry. (**A**) The control group; (**B**) the infection group; (**C**) the Amo20 group; (**D**) the Pro10 group; (**E**) the Pro20 group; (**F**) the Pro40 group; (**G**) the Pro20 + Amo20 group; (**H**) the Bai100 group; (**I**) the results of CD3^+^ T cells in each group. ^###^ *p* < 0.001 versus the control group; * *p* < 0.05, ** *p* < 0.01, and *** *p* < 0.001 versus the infection group.

**Figure 4:**
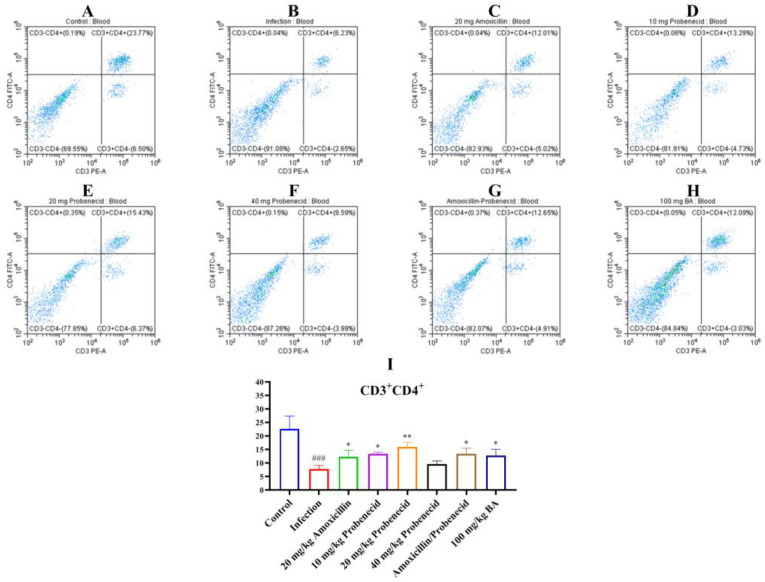
The proportion of CD3^+^CD4^+^ T cells from the blood were analyzed using cytometry. (**A**) The control group; (**B**) the infection group; (**C**) the Amo20 group; (**D**) the Pro10 group; (**E**) the Pro20 group; (**F**) the Pro40 group; (**G**) the Pro20 + Amo20 group; (**H**) the Bai100 group; (**I**) the results of CD3^+^CD4^+^ T cells in each group. ^###^ *p* < 0.001 versus the control group; * *p* < 0.05 and ** *p* < 0.01 versus the infection group.

**Figure 5:**
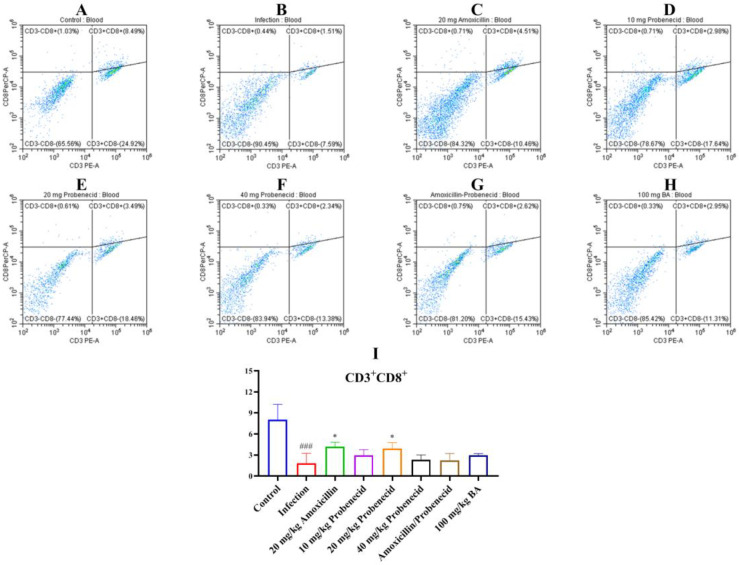
The proportion of CD3^+^CD8^+^ T cells from the blood were analyzed using cytometry. (**A**) The control group; (**B**) the infection group; (**C**) the Amo20 group; (**D**) the Pro10 group; (**E**) the Pro20 group; (**F**) the Pro40 group; (**G**) the Pro20 + Amo20 group; (**H**) the Bai100 group; (**I**) the results of CD3^+^CD8^+^ T cells in each group. ^###^ *p* < 0.001 versus the control group; * *p* < 0.05 versus the infection group.

Immune cells, including T cells, play important roles in regulating innate and adaptive immunity. We used flow cytometry to determine the proportions of T cells in the blood and spleen of *G. parasuis*-challenged mice for 72 h (Figure 3, Figure 4 and Figure 5, Supplemental Figures S1–S3). In the blood, the proportion of CD3^+^, CD3^+^CD4^+^, and CD3^+^CD8^+^ T cells were significantly deceased in the infection group compared with the control group (*p* < 0.001) (Figure 3, Figure 4 and Figure 5). The Pro10, Pro20, Amo20, and Pro20 + Amo20 groups, but not the Pro40 group, showed higher proportions of CD3^+^ and CD3^+^CD4^+^ T cells compared with the infection group (*p* < 0.05) (Figure 3 and Figure 4), while the proportions of CD3^+^CD8^+^ T cells were increased in the Pro20 and Amo20 groups (*p* < 0.05) (Figure 5). When the mice were pretreated with 100 mg/kg BW baicalin, the proportions of CD3^+^ and CD3^+^CD4^+^ T cells were upregulated compared with the infection group (*p* < 0.05) (Figure 3 and Figure 4).

In the spleen, the proportions of CD3^+^, CD3^+^CD4^+^, and CD3^+^CD8^+^ T cells were increased in the infection group compared with the control group (*p* < 0.001) (Supplemental Figures S1–S3). The Pro10, Pro20, Pro40, Amo20, and Pro20 + Amo20 groups showed increased proportions of CD3^+^, CD3^+^CD4^+^, and CD3^+^CD8^+^ T cells compared with the infection group (*p* < 0.01) (Supplemental Figures S1–S3). Finally, 100 mg/kg BW baicalin upregulated the proportions of CD3^+^, CD3^+^CD4^+^, and CD3^+^CD8^+^ T cells compared with the infection group (*p* < 0.001) (Supplemental Figures S1–S3).

**Figure 6:**
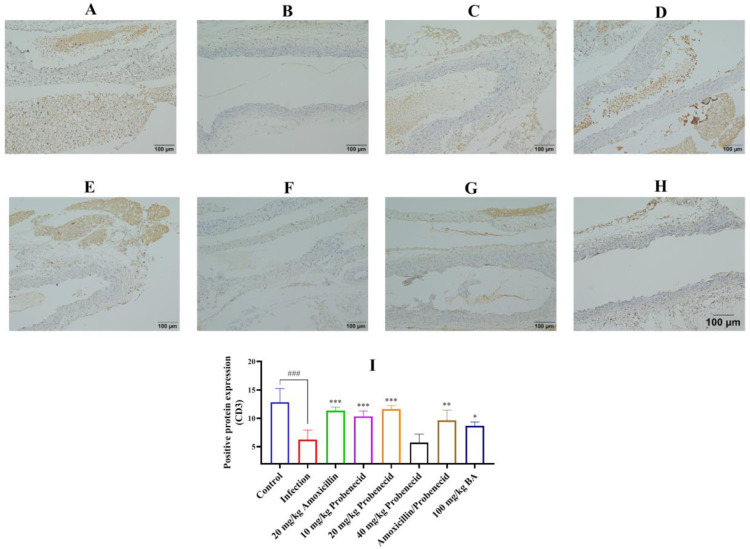
Detection of the CD3 expression in the blood vessels by immunohistochemistry presented in yellow color. Data are expressed as mean ± SD (I). (**A**) the control group; (**B**) the infection group; (**C**) the Amo20 group; (**D**) the Pro10 group; (**E**) the Pro20 group; (**F**) the Pro40 group; (**G**) the Pro20 + Amo20 group; (**H**) the Bai100 group; (**I**) the CD3 expression in the blood vessels. ^###^ *p* < 0.001 versus the control group; * *p* < 0.05, ** *p* < 0.01, and *** *p* < 0.001 versus the infection group.

We also detected the CD3 (Figure 6), CD4 (Supplemental Figure S4), and CD8 (Supplemental Figure S5) expression in the blood vessels by immunohistochemistry. The results showed that the expression of CD3, CD4, and CD8 was intense in the blood vessels of the control group, while in the infection group, the CD3, CD4, and CD8 protein immunostaining was decreased (*p* < 0.001) (Figure 6, Supplemental Figures S4 and S5). Treatment with 20 mg/kg probenecid, 20 mg/kg amoxicillin, the combination of 20 mg/kg probenecid and 20 mg/kg amoxicillin, and 100 mg/kg baicalin could restore the intensity of the CD3, CD4, and CD8 protein expression in the blood vessels of mice compared to the infection group (*p* < 0.05) (Figure 6, Supplemental Figures S4 and S5).

### 2.5. Baicalin, Probenecid, Amoxicillin, and the Combination of Probenecid and Amoxicillin Attenuated NLR Family Pyrin Domain Containing 3 (NLRP3) Inflammasome Activation in Spleen of G. parasuis-Challenged Mice

**Figure 7:**
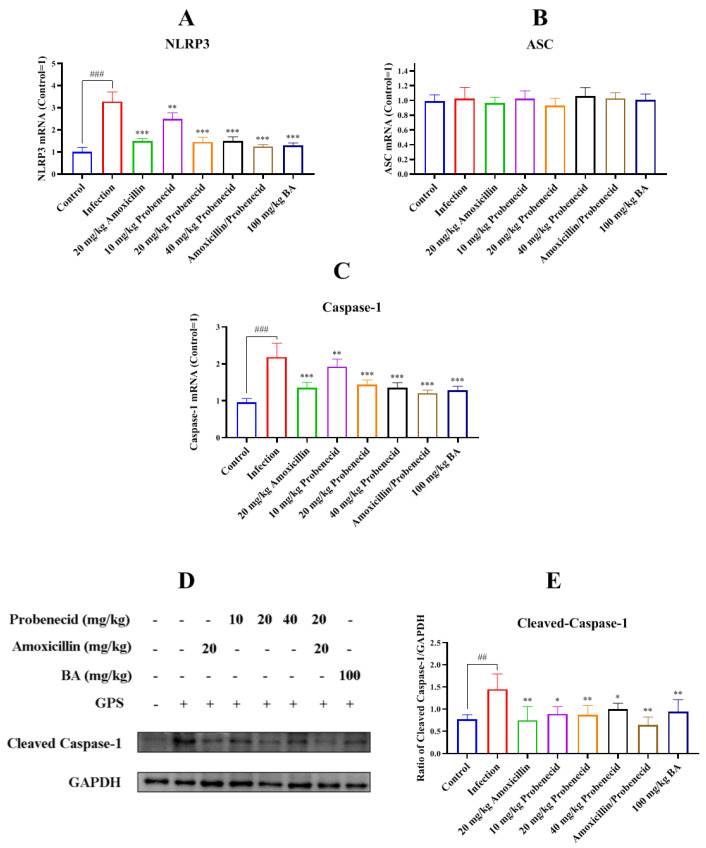
Determination of the effect of baicalin on NLRP3 activation in the spleen of *G. parasuis*-challenged mice. NLRP3 (**A**), ASC (**B**), and caspase-1 (**C**) expressions at mRNA levels were determined by RT-PCR and cleaved caspase-1 protein (**D**,**E**) expression level was evaluated by Western blotting. ^##^ *p* < 0.01 and ^###^ *p* < 0.001 versus the control group; * *p* < 0.05, ** *p* < 0.01, and *** *p* < 0.001 versus the infection group.

We assessed NLRP3 inflammasome activation in the spleen by measuring NLRP3, ASC, and caspase-1 expression. The NLRP3 and caspase-1 mRNA levels were increased in the infection group compared with the control group (*p* < 0.001) (Figure 7A,C). However, the mRNA levels of NLRP3 and caspase-1 were decreased in the Pro10, Pro20, Pro40, Amo20, Pro20 + Amo20, and Bai100 groups compared with the infection group (*p* < 0.01) (Figure 7A,C).

We also used Western blotting to determine the cleaved caspase-1 protein level in the spleen after *G. parasuis* infection. The level was increased in the infection group compared with the control group (*p* < 0.01) (Figure 7D,E), while amoxicillin and probenecid treatment reduced cleaved caspase-1 expression compared with the infection group (*p* < 0.05) (Figure 7D,E). Treatment with 100 mg/kg BW baicalin also attenuated cleaved caspase-1 protein expression compared with the infection group (*p* < 0.01) (Figure 7D,E).

### 2.6. Baicalin, Probenecid, Amoxicillin, and the Combination of Probenecid and Amoxicillin Inhibited Apoptosis in the Spleen of G. parasuis-Challenged Mice

**Figure 8:**
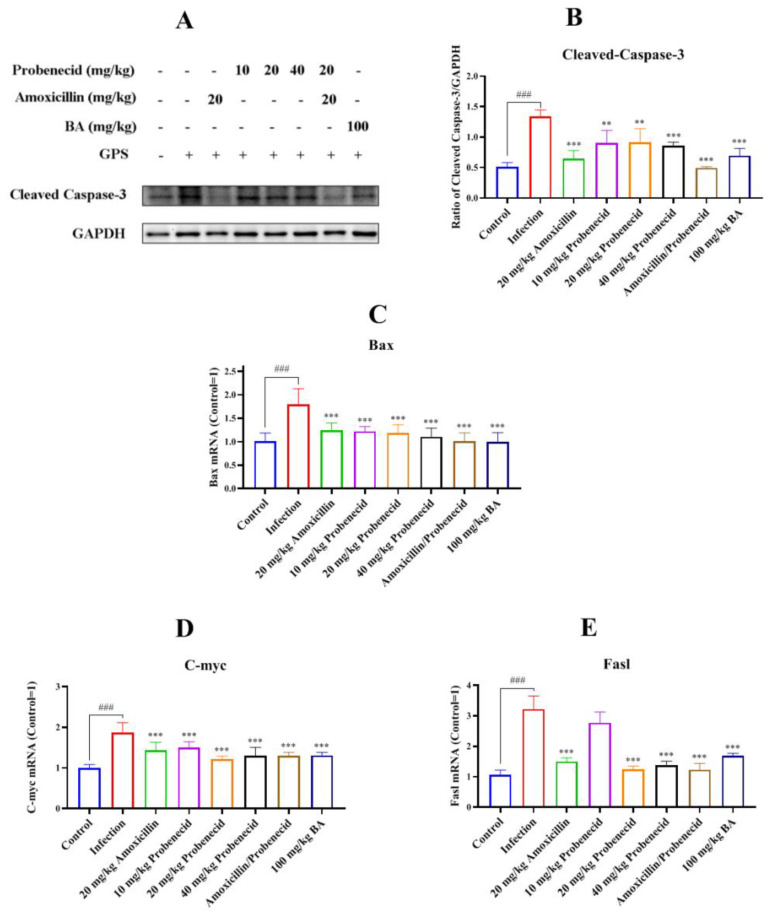
Evaluation of the efficacy of baicalin on cell apoptosis in the spleen of *G. parasuis*-challenged mice. When the mice were infected by *G. parasuis*, the cell apoptosis in the spleen was determined. The activated caspase-3 (**A**,**B**) expression was determined by Western blotting. The Bax (**C**), C-myc (**D**), and Fasl (**E**) expression levels were measured by RT-PCR. ^###^ *p* < 0.001 versus the control group; ** *p* < 0.01 and *** *p* < 0.001 versus the infection group.

To explore the role of caspase-3 in *G. parasuis*-induced apoptosis in the spleen, we used Western blotting to determine the activated caspase-3 protein level. It was increased in the infection group compared with the control group (*p* < 0.001) (Figure 8A,B). Its expression was decreased in Pro10, Pro20, Pro40, Amo20, Pro20 + Amo20, and Bai100 groups compared with the infection group (*p* < 0.01) (Figure 8A,B).

We also assessed the mitochondria-dependent apoptosis pathway induced by *G. parasuis* by examining the mRNA levels of apoptotic genes (Bax, C-myc, and Fasl). The infection group showed elevated Bax, C-myc, and Fasl mRNA levels compared with the control group (*p* < 0.001) (Figure 8C–E). All levels were decreased in the Pro20, Pro40, Amo20, Pro20 + Amo20, and Bai100 groups compared to the infection group (*p* < 0.001) (Figure 8C–E).

### 2.7. Baicalin, Probenecid, Amoxicillin, and the Combination of Probenecid and Amoxicillin Relieved Pathological Lung and Spleen Tissue Damage in G. parasuis-Challenged Mice

**Figure 9:**
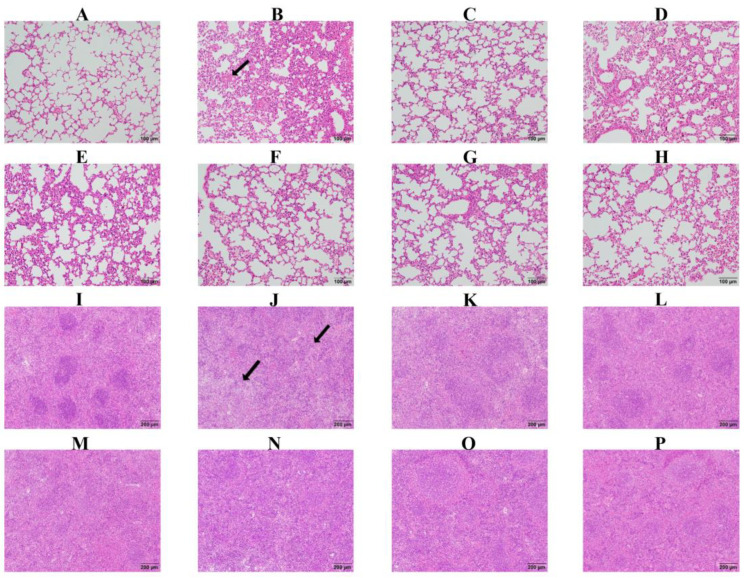
Histopathological analysis of mice lung (**A**–**H**) and spleen (**I**–**P**) in the control group (**A**,**I**), the infection group (**B**,**J**), the Amo20 group (**C**,**K**), the Pro10 group (**D**,**L**), the Pro20 group (**E**,**M**), the Pro40 group (**F**,**N**), the Pro20 + Amo20 group (**G**,**O**), and the Bai100 group (**H**,**P**). Lung and spleen tissues were fixed by immersion and embedded in paraffin. Tissue sections were cut and stained by hematoxylin and eosin, and examined by light microscopy. The arrow represents the pathological change.

We assessed pathological changes in the lung and spleen. In the control group, there was no obvious pathological damage, while in the infection group, there was serious pathological damage, including hemorrhage and inflammatory infiltration in the lung, and necrosis and white pulp lymphocytes reduction in the spleen (Figure 9). In the Pro10, Pro20, Pro40, Amo20, Pro20 + Amo20, and Bai100 groups, there was only minor pathological damage (Figure 9), suggesting that baicalin, amoxicillin, and probenecid relieve pathological tissue damage caused by *G. parasuis* challenge.

## 3. Discussion

In this study, we compared the efficacy of baicalin, probenecid, and amoxicillin to protect against *G. parasuis* infection in mice. We found that these compounds could provide protection against *G. parasuis* infection. Our study is the first report regarding the ability of baicalin and probenecid to protect against *G. parasuis*. These findings might provide a new approach for the control of *G. parasuis* infection through combination therapy, but the use of a mouse model may not fully replicate the conditions of *G. parasuis* infection in pigs; thus, we will use a piglet model to explore the protection of baicalin, amoxicillin, and probenecid in piglets against a *G. parasuis* challenge in the future study.

A bacterial infection can elicit the host’s innate and adaptive immune responses [23]. T cells recognize pathogens, including bacteria, that infect host cells [24]. CD3 is a T cell surface marker [25], and CD4+ T helper cells are key regulators of host health and disease [26]. CD8^+^T cells, known as cytotoxic T cells, are the key component of the host’s adaptive immune system. They are responsible for killing cells infected by bacteria or viruses and form memory T cells to provide long-term protection for the host [27]. In chronic infections (e.g., HIV) and the tumor microenvironment, the auxiliary function of CD4^+^ T cells is dysregulated, and they cannot provide effective assistance to CD8^+^ T cells [28].

Although *G. parasuis* is known as the causative pathogen of Glässer’s disease, it is still unclear how the adaptive immune system responds to this bacterium. *G. parasuis* could elicit immunosuppression, but the exact mechanism remains unclear. We found that the WBC and LYM levels decreased sharply from 12 to 72 h after the *G. parasuis* challenge. We also assessed the CD3^+^, CD3^+^CD4^+^, and CD3^+^CD8^+^ T cell levels in the blood and spleen. The proportion of each population was significantly decreased in the infection group compared with the control group. Previous research reported that a decrease in CD4^+^ T cells reduces macrophage phagocytosis and the ability to kill pathogens [29]. This might be one of the mechanisms by which *G. parasuis* induces immunosuppression. We found that amoxicillin, probenecid, and baicalin could reverse the reduction in CD3^+^, CD3^+^CD4^+^, and CD3^+^CD8^+^ T cell levels. These findings shed light on how the adaptive immune system responds to *G. parasuis* infection, and how amoxicillin, probenecid, and baicalin exert an immunomodulatory effect on T cell differentiation.

HMGB1 is a prototypical damage-associated molecular pattern (DAMP) and promotes inflammation [30]. HMGB1 plays a pivotal role in inflammatory diseases by binding to its corresponding receptors [31]. In mice, HMGB1 enables bacterial lipids to trigger receptor-interacting protein kinase 3 (RIPK3)-mediated necroptosis and apoptosis, leading to tissue damage [32]. HMGB1 is involved in LPS-induced hyperinflammation in mice [33]. The deletion of HMGB1 in airway epithelial cells exaggerates the inflammatory response in mice with muco-obstructive airway disease [34]. We found that *G. parasuis* promoted HMGB1 production in the spleen, which might result in tissue damage, but the mechanism needs to be investigated more deeply. Probenecid provides protection against oxygen–glucose deprivation injury in primary astrocytes by inhibiting HMGB1 production [35]. Probenecid also protects against transient focal cerebral ischemic injury by inhibiting HMGB1 release in mice [36]. Baicalin protected piglets from a *G. parasuis* challenge by reducing HMGB1 production [37]. In the present study, baicalin, probenecid, and amoxicillin attenuated HMGB1 release, which relieved tissue damage, but the exact mechanism requires further elucidation.

The NLRP3 inflammasome is an intracellular multiprotein complex that can be activated by a series of pathogenic microbes [38]. NLRP3 inflammasome activation in bone marrow-derived macrophages leads to liver fibrosis [39]. Moreover, the NLRP3 inflammasome is involved in Alzheimer’s disease-related neuroinflammation [40]. Researchers have reported that the NLRP3 inflammasome can mediate apoptosis [41,42]. We found that *G. parasuis* challenge activated the NLRP3 inflammasome signaling pathway and apoptosis in the spleen.

Many traditional Chinese medicines are anti-inflammatory. Shizhifang inhibits hyperuricemia-induced renal tubular epithelial cell pyroptosis through targeting NLRP3 to induce tubular inflammation [43]. QijiShujiang granules alleviate the dopaminergic neuronal injury of Parkinson’s disease by inhibiting the NLRP3/caspase-1 pathway [44]. Yunvjian decoction attenuates LPS-induced periodontitis in alveolar bone by suppressing the NLRP3 pathway [45]. We found that amoxicillin, probenecid, and baicalin attenuated NLRP3 pathway activation and apoptosis in the spleen triggered by *G. parasuis*.

NLRP3 inflammasome activation results in the release of cytokines such as IL-1β, IL-18, and TNF-α [38]. TNF-α and IL-1β are the key drivers of retinal endothelial dysfunction in non-infectious uveitis, leading to macular oedema and vascular leakage [46]. IL-1β disrupts blood–brain barrier development by inhibiting endothelial Wnt/β-catenin signaling during neuroinflammation [47]. IL-1β induces inflammatory responses in human aortic valve interstitial cells (AVICs) and is involved in the pathogenesis of aortic stenosis [48]. IL-18 induces inflammatory and cytotoxic immune cell activities, contributing to autoimmunity [49]. Exaggerated cytokine responses could cause severe tissue damage [50]. We found that amoxicillin, probenecid, and baicalin could reduce the tissue damage induced by *G. parasuis* and attenuated IL-1β, IL-18, and TNF-α expression.

## 4. Materials and Methods

### 4.1. Ethics Statement

This animal study was approved by the Animal Care and Use Committee of Wuhan Polytechnic University, Hubei Province, China (WPU202307002). All experimental animals were euthanized via intraperitoneal injection with 1% pentobarbital sodium (50 mg/kg) at the end of the experiment.

### 4.2. Bacterial and Culture Conditions

*G. parasuis* SH0165 serovar 5 was originally isolated from the lung of a commercially produced pig that had the typical Glässer’s disease characteristics of arthritis, fibrinous polyserositis, hemorrhagic pneumonia, and meningitis [37]. The SH0165 strain was cultured in tryptic soy broth (TSB) (Difco Laboratories, Detroit, MI, USA) or tryptic soy agar (TSA) (Difco Laboratories) supplemented with 10 μg/mL nicotinamide adenine dinucleotide (NAD) (Ameresco, Framingham, MA, USA) and 10% fetal bovine serum (Sijiqing, Jiangsu Sijiqing Biotechnology Co., Ltd., Nanjing, China) at 37 °C.

### 4.3. Drugs and the Mice

Baicalin was purchased from Sichuan Taikang Pharmaceutical Co., Ltd. (Yuechi, China). When used, baicalin was dissolved and diluted in TSB. Probenecid and amoxicillin were obtained from MedChemexpress (Monmouth Junction, NJ, USA).

One hundred and sixty 4–5-week old female BALB/c mice were purchased from Hubei Provincial Center for Disease Control and Prevention (China).

### 4.4. Experimental Design

The mice were randomly divided into eight groups (20 mice/group): the control group, the infection group, the 10 mg/kg BW probenecid group (Pro10), the 20 mg/kg BW probenecid group (Pro20), the 40 mg/kg BW probenecid group (Pro40), the amoxicillin group (Amo20), the probenecid and amoxicillin group (Pro20 + Amo20), and the baicalin group (Bai100). Before the *G. parasuis* challenge, the mice from the Pro10, Pro20, and Pro40 groups were injected intramuscularly with 10, 20, and 40 mg/kg BW probenecid, respectively. The mice in the Amo20 group were injected intramuscularly with 20 mg/kg BW amoxicillin. The mice in the Pro20 + Amo20 group were injected intramuscularly with 20 mg/kg BW probenecid and 20 mg/kg BW amoxicillin. The mice in the Bai100 group were injected intramuscularly with 100 mg/kg BW baicalin. After 2 h, the infection, Pro10, Pro20, Pro40, Amo20, Pro20 + Amo20, and Bai100 groups were challenged with an intraperitoneal injection of 2 × 10^9^ CFU *G. parasuis* in 1 mL of TSB. The mice from the control group were injected intraperitoneally with an equivalent volume of TSB. The drugs were administered twice a day for 2 days. All mice were monitored for 7 days after the *G. parasuis* challenge, during which time their BW, morbidity, and mortality were recorded.

### 4.5. Blood Biochemical Parameters and Routine Blood Test Indicators

At 12, 24, and 72 h after the *G. parasuis* challenge, blood was collected to assess blood biochemical parameters and routine blood test indicators. The blood biochemical parameters were explored as described in a previous study [51]. Briefly, 500 μL of blood from the caudal vein was collected in the tubes containing ethylenediaminetetraacetic acid (EDTA). The blood samples were centrifuged at 3000 rpm for 30 min under 4 °C to obtain plasma samples, which were analyzed with commercially available kits (Shanghai KehuaBio-engineering Co., Ltd., Shanghai, China). The biochemical parameters detected included TB, total protein (TP), ALB, AST, ALT, ALP, CHOL, TG, GLU, Ca, P, HDL, low-density lipoprotein (LDL), and LDH. Anticoagulated blood samples were used to determine the WBC, RBC, hemoglobin (HGB), PLT, NE, LYM, MONO, and EOS levels with an automatic blood analyzer (Hitachi HITEC 7100, Tokyo, Japan).

### 4.6. Flow Cytometry

Anticoagulated whole blood samples and spleens from mice 72 h after the *G. parasuis* challenge were used to prepare single-cell suspensions. Splenocytes were isolated from spleens as described previously [52]. Briefly, the spleen samples were filtered through strainers (70 μm) to obtain splenocytes, which were rinsed and resuspended in cold phosphate-buffered saline (PBS), before being lysed with lysis buffer. Afterwards, the blood cells and splenocytes were labelled with the following monoclonal antibodies: PE anti-mouse CD3ε, FITC anti-mouse CD4, PerCP/Cyanine5.5 anti-mouse CD8α (BioLegend, San Diego, CA, USA). The samples were analyzed with flow cytometry (Beckman, Shanghai, China).

### 4.7. Bacterial Burden

After challenging the mice with *G. parasuis* for 24 h, the bacterial burden in the liver, spleen, lung, and lymph node were aseptically collected, tissues were homogenized in sterile PBS (1 mL) using a mechanical homogenizer, and serial 10-fold dilutions of the homogenates were prepared in PBS and cultured in triplicate in TSA at 37 °C for 24 h. Bacterial colonies were counted, and colony-forming units (CFUs) per gram of tissue were calculated.

### 4.8. **RT**-qPCR

RT-qPCR was used to determine the expression of inflammatory cytokines (IL-1β, IL-18, and TNF-α) and HMGB1 [53]. Briefly, total RNA was extracted from spleen samples with the TRIzol reagent (Invitrogen, Waltham, MA, USA) and reverse transcribed to complementary DNA (cDNA) using reverse transcriptase (TaKaRa, Beijing, China) according to the manufacturer’s protocol. RT-qPCR was performed by using the SYBR Green PCR Kit (TaKaRa), following the manufacturer’s protocol, and a 7500 Real-Time PCR System (Applied Biosystems, Waltham, MA, USA). The specific amplification of each gene was verified by using melting curve analysis. The relative fold change was calculated by using the 2^−ΔΔCt^ method. The primers used in this study for RT-PCR are presented in Table 4.

### 4.9. Western Blotting

Western blotting was performed as described previously [37] with some minor modifications. Briefly, total protein was extracted from spleens with a total protein extraction kit (Beyotime Biotechnology, Shanghai, China). The protein concentration was determined by a BCA protein assay kit (Sigma, Bellevue, WA, USA). Proteins were resolved with 12% sodium dodecyl sulphate–polyacrylamide gel electrophoresis (SDS-PAGE) and transferred onto polyvinylidene difluoride membranes. The membranes were incubated in 5% non-fat milk for 90 min to block nonspecific protein binding and then washed five times with Tris-buffered saline containing Tween 20 (TBST). The membranes were incubated with primary antibody of IL-1β (Cat. No. A1112, Polyclonal, 1:1000, ABclonal, Woburn, MA, USA), IL-18 (Cat. No. A23076, Monoclonal, 1:5000, ABclonal), TNF-α (Cat. No. 60291-1, Monoclonal, 1:1000, Proteintech, Tokyo, Japan), HMGB1 (Cat. No. PAB12414, Polyclonal, 1:1000, Abnova, Taipei City, Taiwan), cleaved caspase-1 (Cat. No. ABP0172, Polyclonal, 1:1000, Abbkine, Atlanta, GA, USA), activated caspase-3 (Cat. No. 9661S, Monoclonal, 1:1000, Cell Signaling, Danvers, MA, USA), or GAPDH (Cat. No. 10494-1-AP, Polyclonal, 1:5000, Proteintech, Rosemont, IL, USA) for 12 h at 4 °C. After washing the membranes five times with TBST, they were incubated with horseradish peroxidase-linked goat anti-rabbit antibody (Proteintech, Rosemont, IL, USA) for 30 min at 37 °C. The colored bands were analyzed by utilizing ImageJ 1.52a software to measure the gray values with a FluorChemFC2 AIC system (Alpha Innotech, San Leandro, CA, USA). The protein expression levels were measured by comparing the gray values of the colored bands with the gray values of the internal controls.

### 4.10. Immunohistochemistry

Immunohistochemistry was performed as described previously [54] with some minor modifications. Briefly, blood vessel samples from mice were collected 72 h after the *G. parasuis* challenge, and following rehydration in a graded alcohol series, the slides were heated in an appropriate buffer solution and incubated with blocking solution to reduce nonspecific background staining. After incubation with the primary antibody for 1 h, the slides were washed with buffer solution to remove unbound primary antibody. Then, the slides were incubated with secondary antibody for 1 h. After washing in buffer solution, substrate or a fluorophore was added to the slides to visualize the antigen–antibody complex. Following counterstaining with hematoxylin, the slides were dehydrated through a graded alcohol series and cleared with xylene.

### 4.11. Histopathological Analysis

Lung and spleen tissues were fixed by immersion in 10% neutral buffered formalin and then embedded in paraffin. After embedding, 4 μm tissue sections were cut and stained with hematoxylin and eosin using a standard protocol. The stained sections were examined with a light microscope.

### 4.12. Statistical Analysis

The experimental data are presented as the mean ± standard deviation. Statistical differences were determined with analysis of variance (ANOVA). The log-rank test was used for survival analysis. *p* < 0.05 was considered to be a statistically significant difference.

## 5. Conclusions

Taken together, our results demonstrated that amoxicillin, probenecid, and baicalin improved the blood biochemical parameters, routine blood test indicators, and survival rate, and reduced the bacterial burden and pathological tissue damage caused by a *G. parasuis* challenge. These compounds modified CD3+, CD3+CD4+, and CD3+CD8+ T cell differentiation in the blood and splenocytes; attenuated IL-1β, IL-18, TNF-α, and HMGB1 release; and inhibited NLRP3 inflammasome activation and apoptosis in the spleen. Our research provides new insights regarding the combined use of antibiotics and anti-inflammatory agents to control *G. parasuis* infection.

## Figures and Tables

**Table 1:** The blood biochemical parameters for 12 h.

Item	Control	GPS	Amo20	Pro10	Pro20	Pro40	Pro20 + Amo20	Bai100	SEM	*p* Value						
	(A)	(B)	(C)	(D)	(E)	(F)	(G)	(H)		BvsA	CvsB	DvsB	EvsB	FvsB	GvsB	HvsB
TB (mg/dL)	0.13	1.20	0.60	1.00	0.90	0.70	0.40	1.08	0.10	0.005	0.089	0.556	0.378	0.146	0.028	0.734
TP (g/dL)	30.99	30.16	28.40	28.18	33.48	30.97	30.84	29.27	0.48	0.626	0.311	0.255	0.065	0.633	0.689	0.603
ALB (g/dL)	21.73	16.01	17.76	17.36	19.67	18.85	19.41	17.54	0.46	0.001	0.248	0.369	0.024	0.070	0.034	0.309
AST (U/L)	124.00	142.00	113.00	221.00	268.00	344.00	258.00	208.00	17.29	0.621	0.415	0.039	0.002	<0.001	0.005	0.077
ALT (U/L)	26.00	62.00	38.00	40.00	60.00	52.00	50.00	51.00	2.92	<0.001	0.009	0.015	0.872	0.251	0.170	0.221
ALP (U/L)	185.00	97.00	95.00	99.00	76.00	96.00	107.00	106.00	6.80	<0.001	0.881	0.811	0.078	0.928	0.360	0.424
CHOL (mg/dL)	1.19	2.00	1.68	1.74	1.94	1.85	1.83	1.51	0.06	<0.001	0.005	0.015	0.566	0.149	0.098	<0.001
TG (mg/dL)	1.33	3.10	2.02	1.54	3.47	2.23	1.98	2.69	0.20	0.015	0.118	0.030	0.570	0.203	0.106	0.544
GLU (mg/dL)	3.53	0.70	1.03	1.57	0.72	0.83	1.57	1.05	0.19	<0.001	0.302	0.014	0.958	0.676	0.014	0.284
Ca (mg/dL)	2.11	1.55	1.99	1.77	1.54	1.72	1.79	1.56	0.05	<0.001	0.002	0.084	0.956	0.174	0.065	0.956
P (mg/dL)	2.42	1.74	1.73	1.53	1.65	1.69	1.48	1.62	0.07	<0.001	0.053	0.004	0.019	0.032	0.002	0.014
HDL (mg/dL)	0.81	0.76	0.77	0.83	0.89	0.87	0.87	0.71	0.02	0.001	0.985	0.237	0.606	0.788	0.153	0.505
LDL (mg/dL)	0.11	0.07	0.06	0.08	0.10	0.08	0.09	0.08	0.01	0.079	0.777	0.572	0.168	0.777	0.399	0.777
LDH (U/L)	1191.70	620.40	613.10	800.20	1094.70	1159.30	875.60	647.20	53.60	<0.001	0.951	0.142	0.001	<0.001	0.043	0.821

**Table 2:** The blood biochemical parameters for 24 h.

Item	Control	GPS	Amo20	Pro10	Pro20	Pro40	Pro20 + Amo20	Bai100	SEM	*p* Value						
	(A)	(B)	(C)	(D)	(E)	(F)	(G)	(H)		BvsA	CvsB	DvsB	EvsB	FvsB	GvsB	HvsB
TB (mg/dL)	0.17	0.56	0.33	0.62	0.14	0.42	0.26	0.31	0.04	<0.001	0.001	0.246	<0.001	0.022	<0.001	<0.001
TP (g/dL)	32.18	32.15	31.59	32.63	31.97	29.72	29.43	30.09	0.44	0.989	0.750	0.786	0.918	0.179	0.135	0.252
ALB (g/dL)	22.15	16.44	17.51	18.62	18.33	17.67	20.07	19.99	0.41	<0.001	0.311	0.048	0.082	0.245	0.003	0.003
AST (U/L)	119.00	135.00	120.00	159.00	158.00	218.00	196.00	154.00	7.72	0.361	0.402	0.167	0.184	<0.001	0.002	0.265
ALT (U/L)	21.00	34.00	25.00	32.00	38.00	40.00	32.00	35.00	1.51	0.003	0.029	0.615	0.360	0.143	0.615	0.801
ALP (U/L)	183.00	68.00	77.00	102.00	100.00	86.00	103.00	83.00	7.24	<0.001	0.329	0.002	0.002	0.053	0.001	0.099
CHOL (mg/dL)	1.21	1.92	1.57	1.71	1.88	1.62	1.55	1.75	0.05	<0.001	0.018	0.141	0.754	0.042	0.014	0.220
TG (mg/dL)	1.32	1.59	0.90	0.81	1.20	0.98	0.96	1.15	0.07	0.275	0.011	0.005	0.125	0.022	0.019	0.084
GLU (mg/dL)	3.47	1.10	0.93	1.50	0.90	1.10	1.71	1.97	0.22	0.003	0.809	0.564	0.772	1.000	0.382	0.220
Ca (mg/dL)	2.09	1.47	1.31	1.97	1.92	1.68	1.44	2.05	0.07	0.002	0.347	0.007	0.014	0.206	0.855	0.003
P (mg/dL)	2.24	1.01	1.22	1.50	1.37	1.27	1.67	1.22	0.09	<0.001	0.460	0.092	0.210	0.350	0.028	0.460
HDL (mg/dL)	0.70	0.74	0.70	0.76	0.82	0.78	0.81	0.84	0.02	0.968	0.609	0.480	0.179	0.330	0.204	0.111
LDL (mg/dL)	0.12	0.10	0.09	0.10	0.10	0.08	0.08	0.09	0.01	0.274	0.852	0.710	0.710	0.461	0.359	0.852
LDH (U/L)	1221.30	621.20	636.13	820.00	867.20	817.30	855.20	657.50	40.07	<0.001	0.813	0.006	0.001	0.006	0.001	0.568

**Table 3:** The blood biochemical parameters for 72 h.

Item	Control	GPS	Amo20	Pro10	Pro20	Pro40	Pro20 + Amo20	Bai100	SEM	*p* Value						
	(A)	(B)	(C)	(D)	(E)	(F)	(G)	(H)		BvsA	CvsB	DvsB	EvsB	FvsB	GvsB	HvsB
TB (mg/dL)	0.15	0.57	0.18	0.25	0.21	0.41	0.29	0.22	0.03	<0.001	<0.001	<0.001	<0.001	0.045	0.002	<0.001
TP (g/dL)	31.07	28.84	31.39	30.91	30.36	30.00	29.14	30.38	0.36	0.150	0.104	0.181	0.319	0.441	0.839	0.312
ALB (g/dL)	20.09	15.02	17.01	16.68	17.39	18.19	18.37	19.76	0.39	<0.001	0.075	0.133	0.038	0.008	0.006	<0.001
AST (U/L)	123.00	84.00	119.00	106.00	117.00	128.00	107.00	110.00	3.62	0.004	0.007	0.073	0.011	0.002	0.066	0.036
ALT (U/L)	21.00	20.00	13.00	20.00	16.00	23.00	12.00	14.00	0.95	0.622	0.030	1.000	0.227	0.274	0.008	0.061
ALP (U/L)	197.00	59.00	65.00	75.00	66.00	53.00	76.00	87.00	10.65	<0.001	0.813	0.544	0.784	0.823	0.520	0.304
CHOL (mg/dL)	1.25	1.08	1.29	1.44	1.48	1.50	1.40	1.53	0.03	0.051	0.021	<0.001	<0.001	<0.001	0.001	<0.001
TG (mg/dL)	1.29	0.53	0.85	0.76	0.81	0.85	0.78	0.84	0.04	<0.001	<0.001	0.004	0.001	<0.001	0.002	<0.001
GLU (mg/dL)	3.44	1.23	1.30	1.87	1.43	1.60	1.33	1.23	0.16	<0.001	0.776	0.011	0.377	0.115	0.656	1.000
Ca (mg/dL)	2.14	2.29	1.96	1.50	2.03	1.90	1.89	1.93	0.06	0.379	0.058	<0.001	0.130	0.028	0.023	0.041
P (mg/dL)	2.31	1.11	1.18	1.38	1.09	1.04	1.26	2.08	0.11	<0.001	0.778	0.307	0.959	0.808	0.566	0.002
HDL (mg/dL)	0.75	0.45	0.52	0.58	0.63	0.57	0.56	0.60	0.02	<0.001	0.256	0.063	0.014	0.077	0.111	0.029
LDL (mg/dL)	0.10	0.15	0.23	0.22	0.23	0.22	0.20	0.21	0.01	0.073	0.014	0.023	0.011	0.018	0.073	0.058
LDH (U/L)	1435.10	755.10	1130.20	805.30	1069.60	1022.30	723.00	895.00	56.03	<0.001	0.025	0.745	0.055	0.097	0.835	0.368

**Table 4:** Primer sequences for qRT-PCR analysis.

Gene	Nucleotide Sequence (5′–3′)		Tm (°C)	Length (bp)
NLRP3	Forward	TTTATTTGTACCCAAGGCTGCTATC	54.7	148
	Reverse	CAACGGACACTCGTCATCTTCA	57.1	
ASC	Forward	CAGTTCGTGCAGAGACCACCA	60.2	140
	Reverse	CTGCTCCAGGTCCATCACCA	60.0	
Caspase-1	Forward	TCCGAGGGTTGGAGCTCAAG	59.9	137
	Reverse	CTGGCCAGGCAGCAAATTC	57.8	
HMGB1	Forward	ATGACAAGCAGCCCTATGAGAA	55.9	82
	Reverse	CCTTTAGCTCTGTAGGCAGCAA	57.1	
Fasl	Forward	GCCATCACAACCACTCCC	56.8	107
	Reverse	CCAGAGCCACCAGAACCA	57.9	
Bax	Forward	AGCTGCAGAGGATGATTGCTG	57.9	178
	Reverse	CTGATCAGCTCGGGCATTTA	54.8	
C-myc	Forward	TCGCCCAAATCCTGTACCTC	57.2	193
	Reverse	TCTCCACAGACACCACATCAAT	55.8	
IL-1β	Forward	TGCCACCTTTTGACAGTGATG	55.6	138
	Reverse	TGATGTGCTGCTGCGAGATT	57.3	
IL-18	Forward	GGACACTTTCTTGCTTGCCA	56.0	166
	Reverse	CAGCCTCGGGTATTCTGTTATG	55.4	
TNF-α	Forward	GCCCCCAGTCTGTATCCTTCTA	58.4	209
	Reverse	TTCGGAAAGCCCATTTGAGT	54.3	
GAPDH	Forward	AAGCCCATCACCATCTTCCA	56.4	88
	Reverse	CACCAGTAGACTCCACGACA	56.4	

## Data Availability

All relevant data are within the manuscript.

## Supplementary Materials

The following supporting information can be downloaded at https://www.mdpi.com/article/10.3390/biom15040507/s1.
