# Supplementary material for: Baicalin, Amoxicillin, and Probenecid Provide Protection in Mice Against Glaesserella parasuis Challenge
Source: Biomolecules. 2025 Mar 31;15(4):507. doi: 10.3390/biom15040507 (PMC12024593; doi:10.3390/biom15040507)
Supplement: Supplementary file 1 [file biomolecules-15-00507-s001.zip › Supplemental Table S1.pdf]

Supplemental Table S1. The routine blood test indicators for 12 h

Item	Control（A）	GPS（B）	Amo20（C）	Pro10（D）	Pro20（E）	Pro40（F）	Pro20+Amo20（G）	Bai100（H）	SEM	*p* value						
										BvsA	CvsB	DvsB	EvsB	FvsB	GvsB	HvsB
WBC（10^9/L）	8.39	1.35	1.67	1.73	3.26	1.68	2.43	1.80	0.41	＜0.001	0.717	0.919	0.009	0.833	0.107	0.219
RBC（10^9/L）	6.74	4.55	5.92	5.69	7.32	6.64	6.63	6.23	0.12	0.011	0.279	0.139	0.001	0.003	0.022	0.036
HGB（g/L）	106.00	112.00	96.00	90.00	79.00	101.00	98.00	95.00	2.46	1.000	0.408	0.010	0.094	0.634	0.453	0.43
PLT（10^9/L）	446.00	56.00	88.00	75.00	66.00	98.00	101.00	77.00	27.76	＜0.001	0.009	0.487	0.737	0.102	0.025	0.144
NE（10^9/L）	0.76	0.51	0.66	1.31	1.01	0.53	0.76	0.72	0.04	0.114	0.630	0.114	0.067	0.374	0.630	0.982
LYM（10^9/L）	4.94	0.90	0.90	1.47	2.11	0.74	1.22	1.86	0.29	＜0.001	0.709	0.992	0.458	0.341	0.626	0.440
MONO（10^9/L）	0.13	0.07	0.25	0.57	0.65	0.36	0.51	0.30	0.03	0.592	0.120	0.005	＜0.001	0.015	0.001	0.025
EOS（10^9/L）	0.18	0.03	0.06	0.05	0.07	0.13	0.13	0.16	0.01	＜0.001	0.335	0.696	0.25	0.002	0.001	＜0.001
