# Supplementary material for: Baicalin, Amoxicillin, and Probenecid Provide Protection in Mice Against Glaesserella parasuis Challenge
Source: Biomolecules. 2025 Mar 31;15(4):507. doi: 10.3390/biom15040507 (PMC12024593; doi:10.3390/biom15040507)
Supplement: Supplementary file 1 [file biomolecules-15-00507-s001.zip › Supplemental Table S2.pdf]

Supplemental Table S2. The routine blood test indicators for 24 h

Item	Control（A）	GPS（B）	Amo20（C）	Pro10（D）	Pro20（E）	Pro40（F）	Pro20+Amo20（G）	Bai100（H）	SEM	*p* value						
										BvsA	CvsB	DvsB	EvsB	FvsB	GvsB	HvsB
WBC（10^9/L）	7.39	1.68	2.97	2.31	2.49	2.82	2.61	2.81	0.16	＜0.001	0.003	0.069	0.015	0.007	0.057	0.013
RBC（10^9/L）	6.95	4.52	6.00	6.16	6.02	6.49	6.48	6.07	0.12	＜0.001	＜0.001	＜0.001	0.001	＜0.001	0.007	＜0.001
HGB（g/L）	117.00	81.00	102.00	105.00	103.00	100.00	95.00	105.00	2.20	＜0.001	＜0.001	＜0.001	＜0.001	＜0.001	0.005	＜0.001
PLT（10^9/L）	440.00	51.00	78.00	156.00	175.00	75.00	69.00	78.00	35.36	＜0.001	0.263	＜0.001	＜0.001	0.305	0.434	0.263
NE（10^9/L）	0.70	1.93	0.48	0.31	0.24	0.44	0.45	0.33	0.08	＜0.001	＜0.001	＜0.001	＜0.001	0.001	＜0.001	＜0.001
LYM（10^9/L）	4.17	0.91	1.74	1.41	1.21	1.41	1.63	1.42	0.17	＜0.001	0.005	0.010	0.048	0.008	0.006	0.015
MONO（10^9/L）	0.14	0.41	0.23	0.23	0.17	0.09	0.08	0.22	0.03	＜0.001	0.001	0.001	＜0.001	＜0.001	＜0.001	0.002
EOS（10^9/L）	0.15	0.05	0.13	0.18	0.28	0.15	0.1	0.12	0.01	0.001	0.004	＜0.001	＜0.001	0.001	0.419	0.037
