# Supplementary material for: Baicalin, Amoxicillin, and Probenecid Provide Protection in Mice Against Glaesserella parasuis Challenge
Source: Biomolecules. 2025 Mar 31;15(4):507. doi: 10.3390/biom15040507 (PMC12024593; doi:10.3390/biom15040507)
Supplement: Supplementary file 1 [file biomolecules-15-00507-s001.zip › Supplemental Table S3.pdf]

**Supplemental Table S3.** The routine blood test indicators for 72 h

| Item | Control（A） | GPS（B） | Amo20（C） | Pro10（D） | Pro20（E） | Pro40（F） | Pro20+Amo20（G） | Bai100（H） | SEM | *p* value | | | | | | |
|---|---|---|---|---|---|---|---|---|---|---|---|---|---|---|---|---|
| | | | | | | | | | | BvsA | CvsB | DvsB | EvsB | FvsB | GvsB | HvsB |
| WBC（$10^9$/L） | 7.88 | 2.18 | 7.22 | 5.89 | 5.56 | 4.18 | 4.22 | 5.29 | 0.31 | ＜0.001 | ＜0.001 | ＜0.001 | ＜0.001 | 0.003 | 0.003 | ＜0.001 |
| RBC（$10^9$/L） | 6.77 | 4.27 | 7.24 | 6.04 | 5.52 | 5.52 | 5.69 | 6.72 | 0.21 | ＜0.001 | ＜0.001 | 0.001 | 0.014 | 0.014 | 0.006 | ＜0.001 |
| HGB（g/L） | 106.00 | 128.00 | 107.00 | 89.00 | 91.00 | 84.00 | 74.00 | 92.00 | 3.37 | ＜0.001 | ＜0.001 | ＜0.001 | ＜0.001 | ＜0.001 | ＜0.001 | ＜0.001 |
| PLT（$10^9$/L） | 431.00 | 78.00 | 221.00 | 145.00 | 174.00 | 126.00 | 397.00 | 623.00 | 39.00 | ＜0.001 | 0.021 | 0.253 | 0.106 | 0.402 | ＜0.001 | ＜0.001 |
| NE（$10^9$/L） | 0.69 | 0.25 | 0.75 | 0.88 | 0.64 | 0.47 | 1.27 | 0.39 | 0.07 | 0.026 | ＜0.001 | ＜0.001 | 0.001 | 0.034 | ＜0.001 | 0.146 |
| LYM（$10^9$/L） | 4.61 | 2.07 | 5.9 | 5.55 | 4.61 | 3.44 | 3.68 | 4.26 | 0.29 | 0.005 | ＜0.001 | ＜0.001 | 0.005 | 0.099 | 0.056 | 0.013 |
| MONO（$10^9$/L） | 0.13 | 0.18 | 0.34 | 0.27 | 0.20 | 0.20 | 0.25 | 0.19 | 0.02 | 0.245 | 0.003 | 0.059 | 0.657 | 0.767 | 0.133 | 0.824 |
| EOS（$10^9$/L） | 0.16 | 0.30 | 0.21 | 0.18 | 0.11 | 0.09 | 0.17 | 0.21 | 0.01 | ＜0.001 | 0.003 | ＜0.001 | ＜0.001 | ＜0.001 | ＜0.001 | 0.003 |
