# Supplementary figures and images for: Baicalin, Amoxicillin, and Probenecid Provide Protection in Mice Against Glaesserella parasuis Challenge
Source: Biomolecules. 2025 Mar 31;15(4):507. doi: 10.3390/biom15040507 (PMC12024593; doi:10.3390/biom15040507)

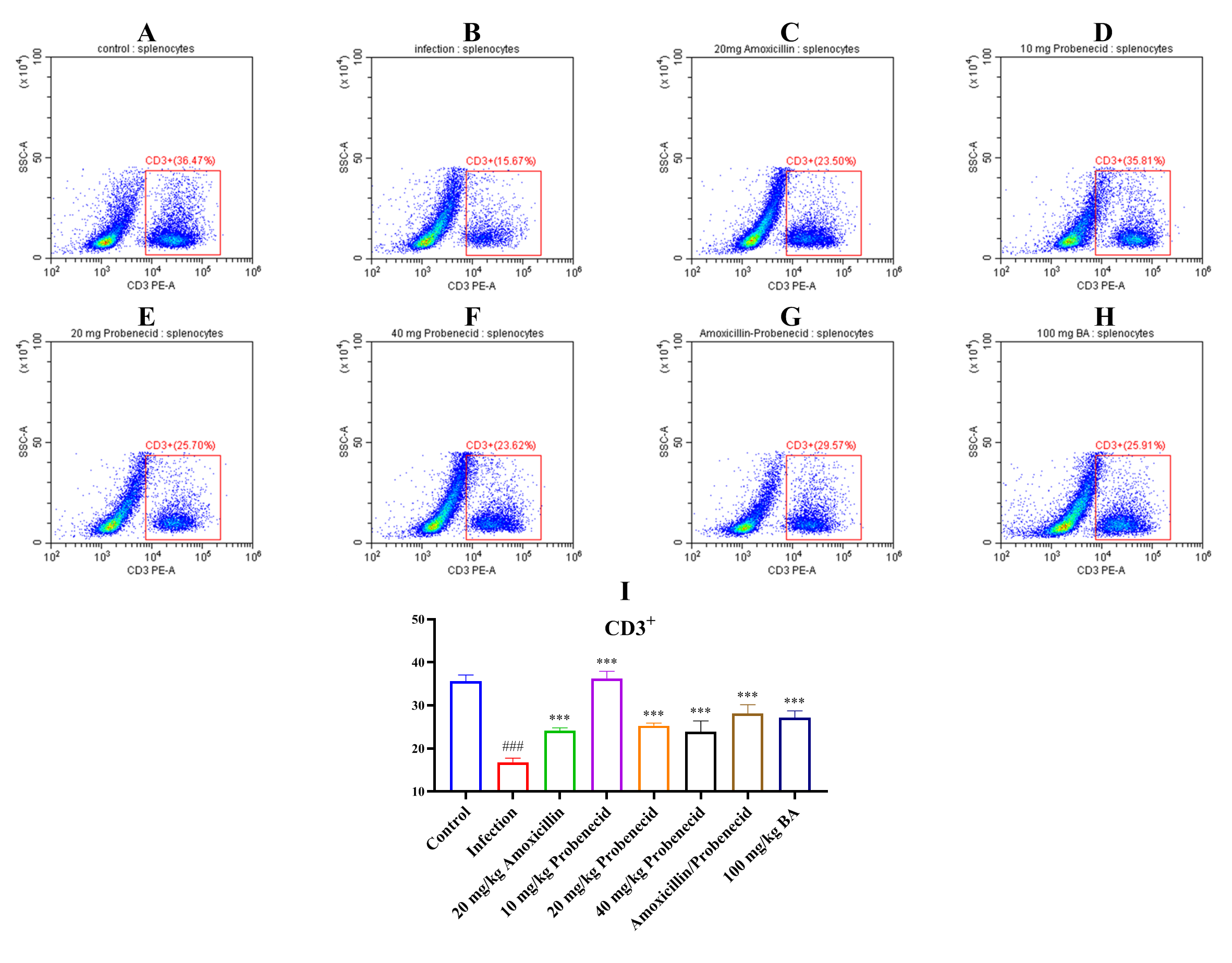

Supplement: Supplementary file 1 [file biomolecules-15-00507-s001.zip › Supplemental Figure S1.tif]

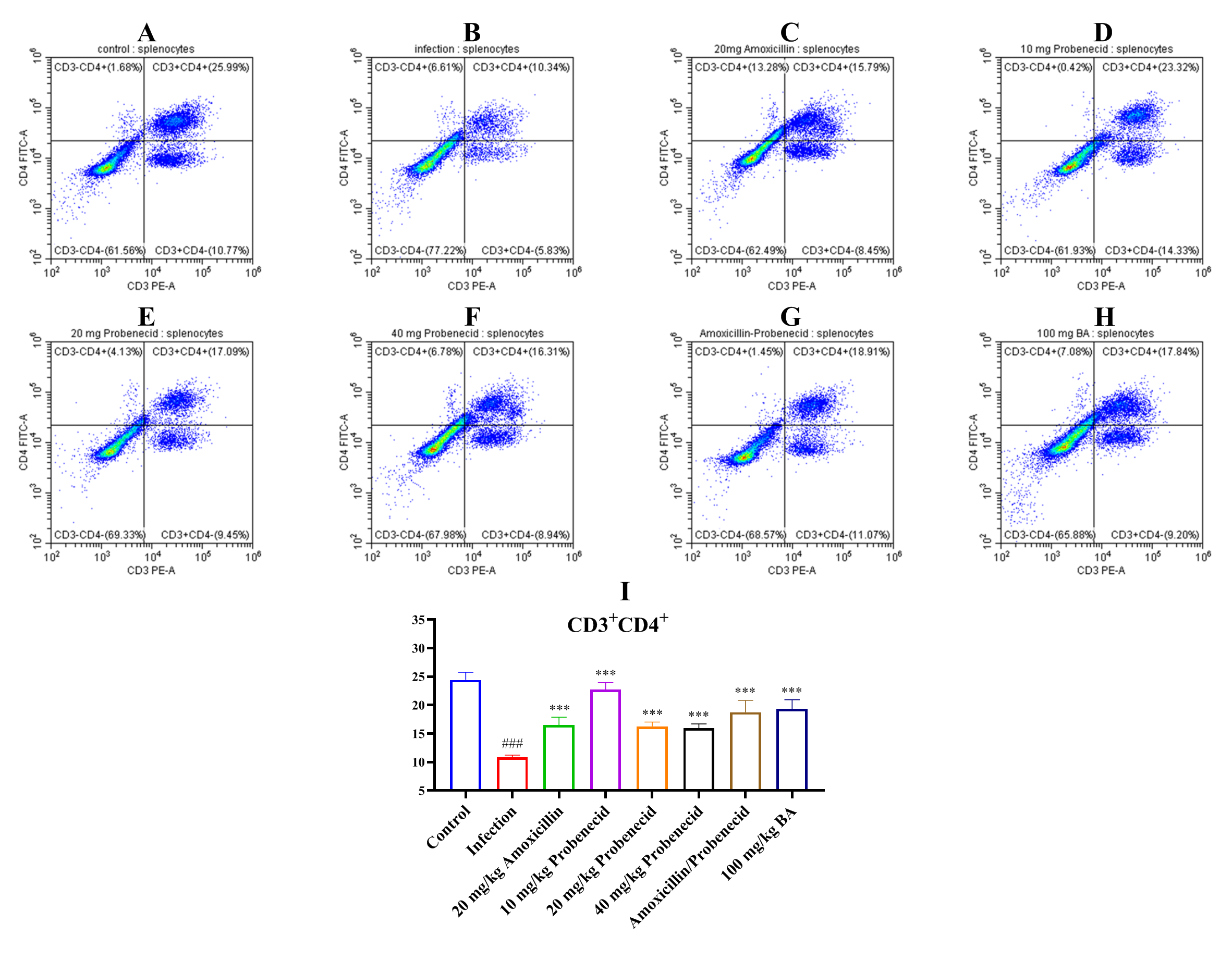

Supplement: Supplementary file 1 [file biomolecules-15-00507-s001.zip › Supplemental Figure S2.tif]

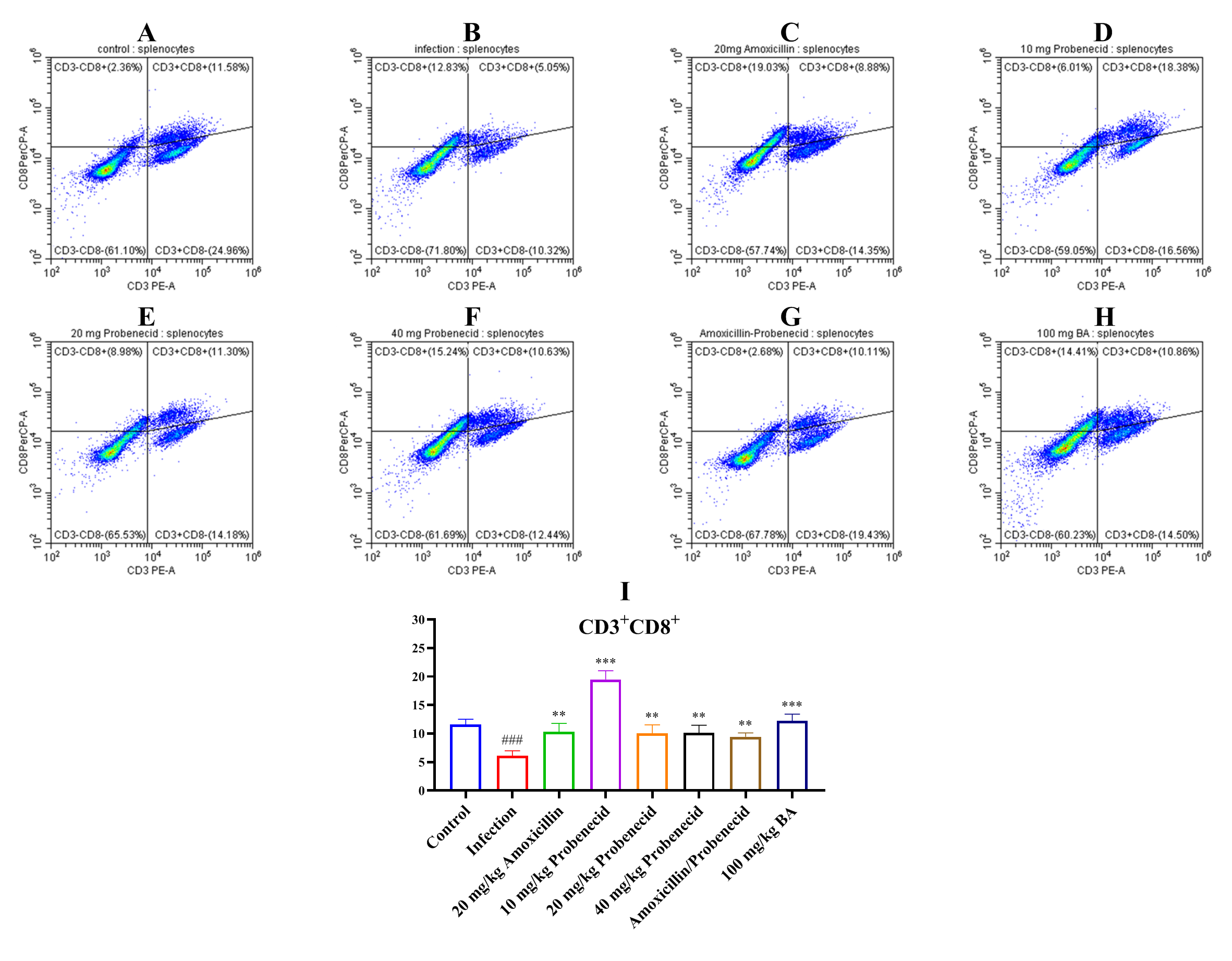

Supplement: Supplementary file 1 [file biomolecules-15-00507-s001.zip › Supplemental Figure S3.tif]

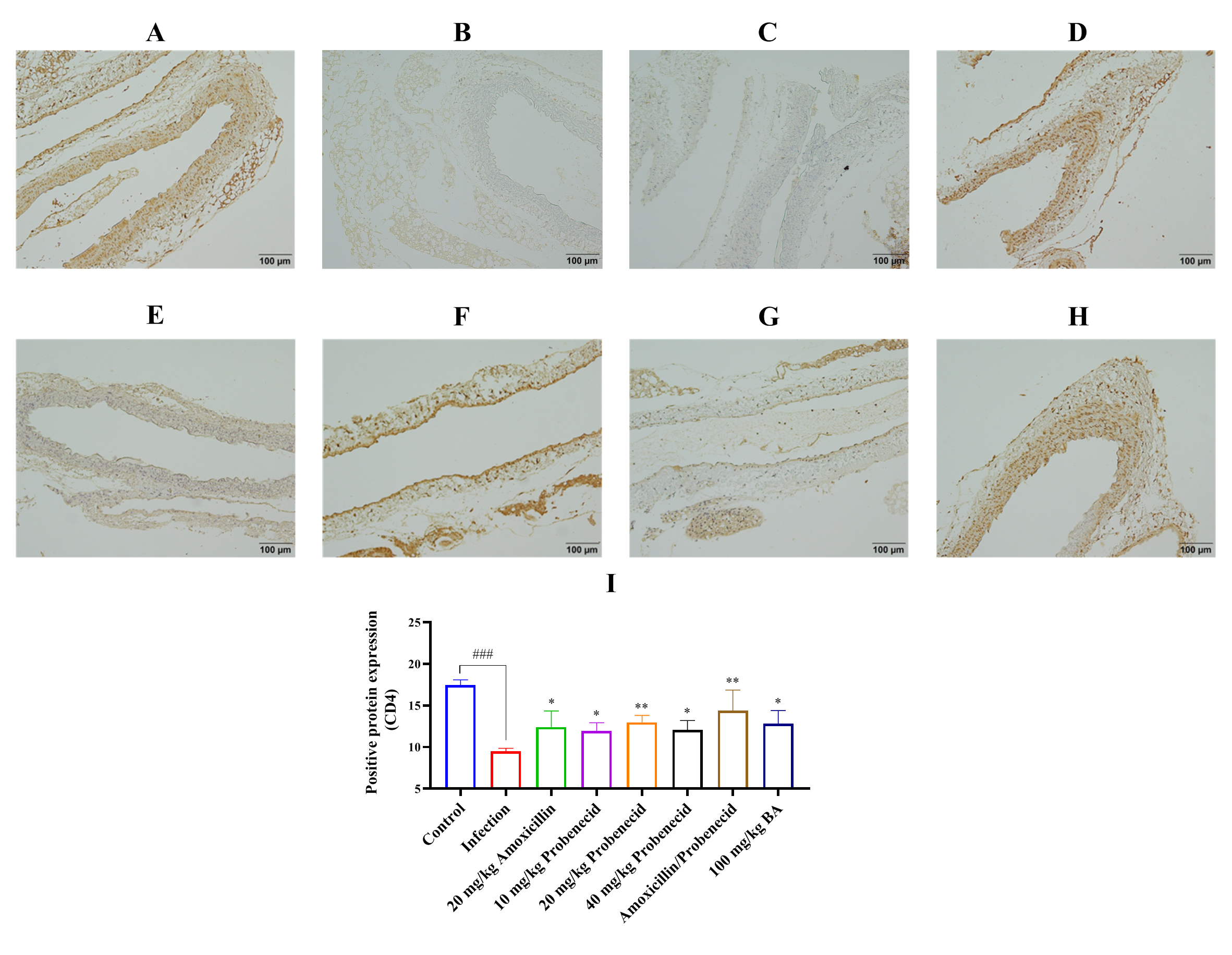

Supplement: Supplementary file 1 [file biomolecules-15-00507-s001.zip › Supplemental Figure S4.tif]

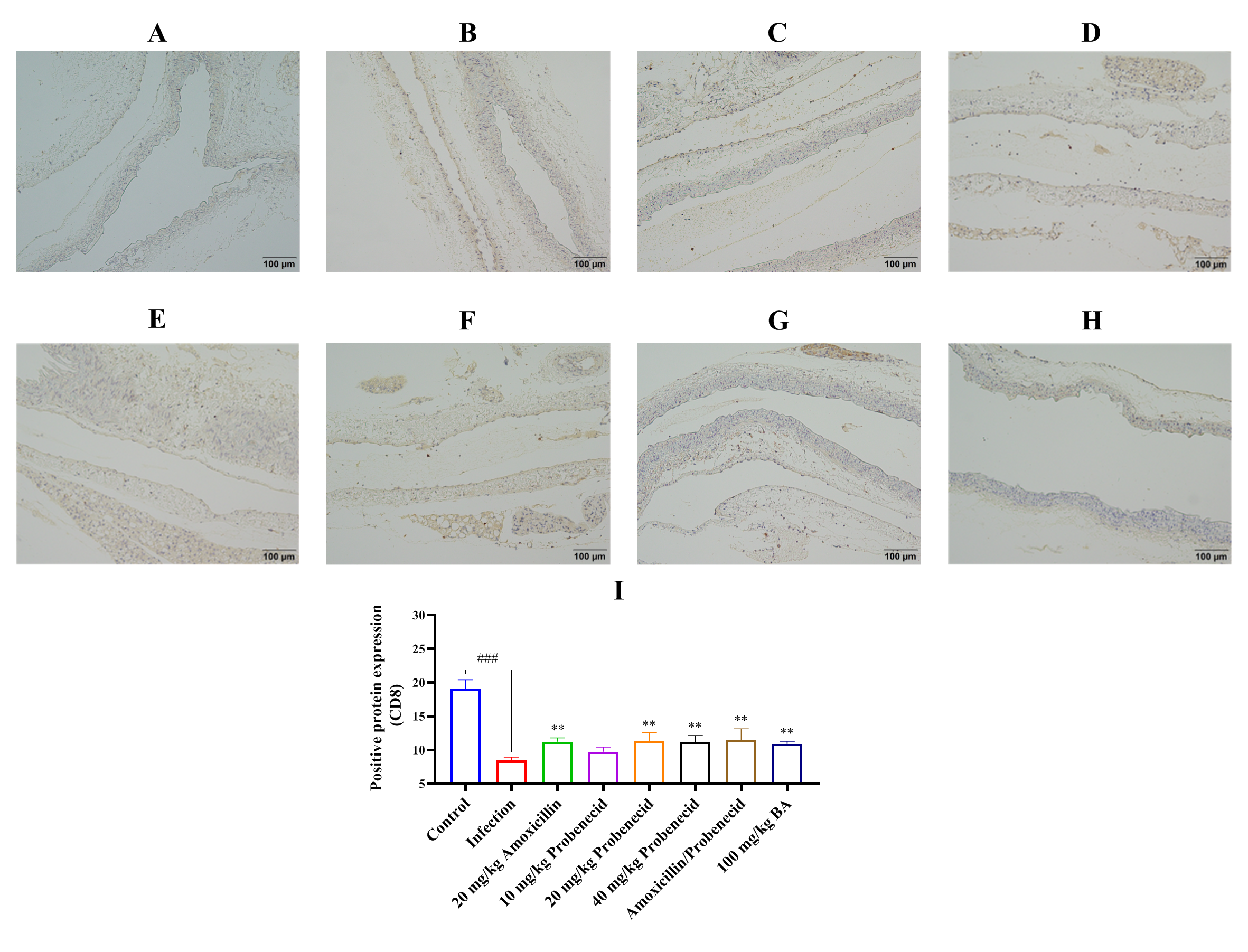

Supplement: Supplementary file 1 [file biomolecules-15-00507-s001.zip › Supplemental Figure S5.tif]
